# Monitoring and Reconstruction of the Shape of the Detection Units in KM3NeT Using Acoustic and Compass Sensors †

**DOI:** 10.3390/s20185116

**Published:** 2020-09-08

**Authors:** Dídac D.Tortosa

**Affiliations:** Institut d’Investigació per a la Gestió Integrada de les Zones Costaneres (IGIC), Universitat Politècnica de València (UPV), Gandia, 46730 València, Spain; didieit@upv.es; Tel.: +34-963-870-000 (ext. 43681)

**Keywords:** underwater acoustics, sensors positioning, KM3NeT

## Abstract

The KM3NeT underwater neutrino telescope comprises thousands of optical modules forming 3D arrays to detect the Cherenkov light produced by particles generated after a neutrino interaction in the medium. The modules are arranged in detection units—vertical structures with 18 modules at different heights, anchored to the seabed and kept vertical by the buoyancy of the optical modules and a top buoy. The optical modules are, thus, subject to movements due to sea currents. For a correct reconstruction of events detected by the telescope, it is necessary to know the relative position and orientation of modules with 10 cm and a few degrees accuracy, respectively. For this, an Acoustic Positioning System with a piezoceramic transducer installed in each module and a long baseline of acoustic transmitters and receivers on the seabed are used. In addition, there is a system of compass and accelerometers inside the optical modules to determine their orientation. A model of mechanical equations is used to reconstruct the shape of the detection unit taking as input the information from the positioning/orientation sensors and using the sea current velocity and direction as free parameters. The mechanical equations take the buoyancy and the drag force of the elements of the detection unit into account. This work describes the full process that is implemented in KM3NeT to monitor the modules and the shape of the detection units from the measured position and orientation data.

## 1. Introduction

**Figure 1:**
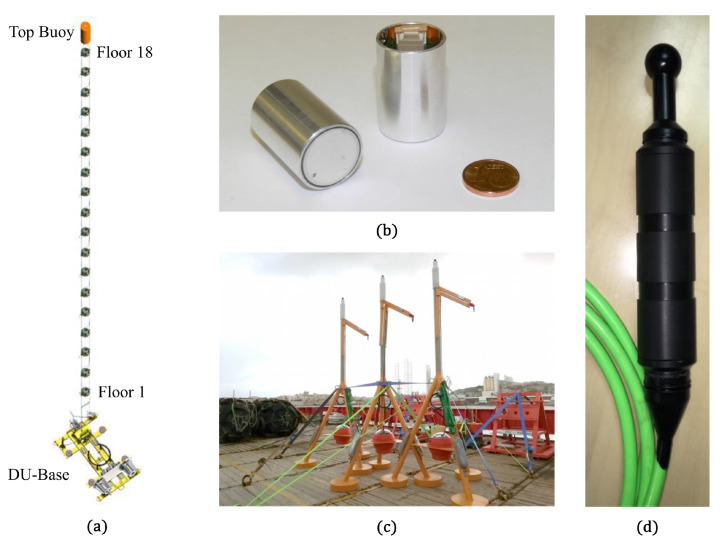
(**a**) Scheme of the detection unit in the KM3NeT detector. It is composed of a detection unit (DU)-base fixed to the anchor with a hydrophone installed among other instruments, 18 Digital Optical Modules (DOMs) (labeled Floor 1, ..., Floor 18), and the top buoy. (**b**) Encapsulated piezoceramic and electronic board with a pre-amplifier. It is the acoustic receiver installed at the bottom of each DOM. (**c**) Three autonomous Acoustic Beacons before their deployment at the Astroparticle Research with Cosmics in the Abyss (ARCA) site. (**d**) Hydrophone installed on the DU-bases of KM3NeT.

The underwater neutrino telescope KM3NeT is under construction in the Mediterranean Sea aiming at a high-energy neutrino detector with high sensitivity. KM3NeT follows in the footsteps of ANTARES, which was the first underwater neutrino telescope in the Mediterranean Sea [1,2]. KM3NeT comprises arrays of Digital Optical Modules (DOMs) to detect the Cherenkov light generated by charged particles produced in neutrino interactions in the water [3]. A detection unit (DU) in KM3NeT is a vertical structure with a top buoy, anchored at the seabed and supporting 18 DOMs (Figure 1a).

**Figure 2:**
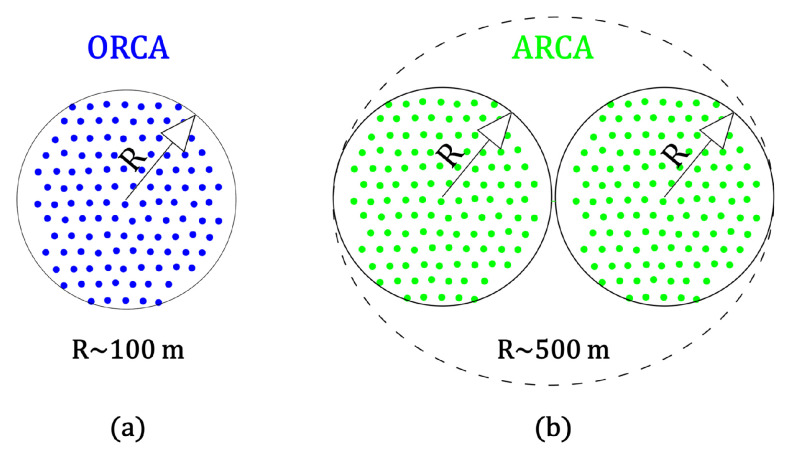
Footprint of KM3NeT at the sea bed. (**a**) The block of Oscillation Research with Cosmics in the Abyss (ORCA) has a radius of about 100 m and has 115 DUs. (**b**) ARCA is composed of two blocks of 115 DUs, each with a radius of about 500 m.

KM3NeT is composed of two different detectors with different scientific aims (Figure 2a). The bigger is the Astroparticle Research with Cosmics in the Abyss (ARCA), located 100 km offshore from Portopalo di Capo Passero at a depth of 3500 m. ARCA is optimized for the search for high-energy cosmic neutrinos. It will be composed of two different detector blocks of 115 DUs with 690 m height. Its instrumented volume will be about one cubic kilometer [4]. The other detector of KM3NeT is Oscillation Research with Cosmics in the Abyss (ORCA), which is located 40 km offshore from Toulon at 2500 m of depth. This detector will contain 115 DUs with a height of 197 m. ORCA is optimized for the study of the properties of neutrinos [5].

Due to sea currents the DU-lines are in motion which causes the positions of the DOMs to vary. To reconstruct a neutrino interaction and its direction, it is crucial to know the DOM positions at the time of the detection with a precision of a few centimeters. Since GPS signals are not available on the ocean depth, the underwater neutrinos telescopes need a positioning system to monitor the position of DOMs in any time. For example, in the Baikal-GVD detector a Hydroacoustic Positioning System is used [6,7], and for the ANTARES detector a line shape model is used to simultaneously fit the data of the Acoustic Positioning System (APS) and the data from the compass-tiltmeter system [8].

KM3NeT positioning relies on the ANTARES line shape model, thanks to the demonstrated improvement [8]. KM3NeT uses an APS to measure the DOMs location and monitors the DOM orientations using an Attitude and Heading Reference System (AHRS). Both information systems help to monitor the location of the DOMs, but for an accurate measurement of the DOMs location data filtering and processing are necessary. This process applied to the APS and AHRS data is called “DU Line Fit”.

The DU Line Fit uses a mechanical model (MM) of mechanical equations using the information from the APS and AHRS systems to reconstruct the shape of the DU line. The aim of the DU Line Fit is to reconstruct the position of the DOMs with a precision of a few centimeters.

This article explains in detail how to obtain a good reconstruction of the shape of DUs in KM3NeT from raw position and orientation data; it justifies the filtering procedure and describes the mechanical model application.

For KM3NeT the DU Line Fit applications is validated by previous studies: the APS to location the DOMs [9], the simultaneously acquisition data by AHRS and APS [10], and the expected improvement of few centimeters in the MM implementation [11].

At this moment, ORCA has six DUs deployed and in operation. For ARCA, two DUs are deployed which currently are out of operation due to an upgrade of the seafloor network.

## 2. Methods

The DU Line Fit procedure is composed of three different parts: the acoustic part (APS), the compass part (AHRS), and the mechanical model part (MM). The DU Line Fit uses APS and AHRS raw data and uses the MM to analyze, process, and reconstruct the shape of the DU. In this section, all parts are explained in detail.

The instrumentation used in APS and AHRS is calibrated before deployment. For example, the acoustic emitters used in APS and the Printed Circuit Board (PCB) used in AHRS are calibrated before installation [10,12].

### 2.1. Acoustic Positioning System (APS)

The Acoustic Positioning System (APS) is focused on determining the position of every DOM. The APS provides the acoustic receiver position, but since its position is fixed and the orientation of DOM is also monitored, the position of the center of the DOM can be determined. The APS consists of acoustic emitters and receivers spread over the full detector. The emitters are Acoustic Beacons (ABs). Currently, ARCA and ORCA have three autonomous ABs each; the maximum distance between a DU-base and the ABs is less than 240 m. KM3NeT has two different types of acoustic receivers: a hydrophone installed on each DU-base and piezoceramics at the bottom inside every DOM. The APS uses the frequency range between 20 and 40 kHz [12].

The APS uses a triangulation method for positioning the receivers. To do this, it is necessary to know where the emitters are anchored and to know the Time of Emission (ToE). The accuracy of deployment location of the DU-bases and the ABs is about 2 m (absolute positioning system), but the accuracy of the relative APS on KM3NeT is few tens of cm [13,14].

#### 2.1.1. Instrumentation in the APS

**Figure 3:**
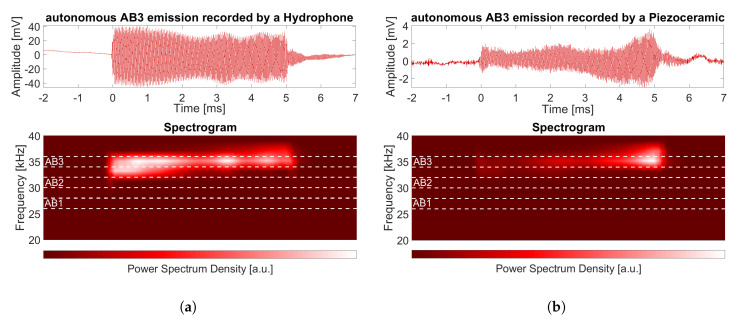
Pulse signal received (time scale is relative to detection time) by different DUs. The distinction of the Acoustic Beacon (AB) emitter is clearly observed in the spectrogram. (**a**) The AB pulse recorded by a hydrophone and its spectrogram. (**b**) The AB pulse recorded by a piezoceramic sensor in the lowest DOM (Floor 1 in Figure 1a) and its spectrogram.

The ABs used by KM3NeT are autonomous, which means that the ToE is not controlled by the system. The autonomous ABs are mounted in tripods with a height of 5 m (see Figure 1c). They are programmed to emit acoustic waves for 1 min and switched off during 9 min after that. During the minute of emissions, the AB emits 1 pulse signal every 5 s. The pulse has a duration of 5 ms, it is a linear sweep-signal along 2 kHz in the frequency band of the emitter (see Figure 3). Each AB has a unique sweep signal that permits distinguishing and detecting it in the reception. The AB’s Sound Pressure Level (SPL) is around 180 dB (re 1 μPa/V at 1 m) in the 20–60 kHz range which allows for a good reception in the receivers [12].

Every DU-base has a hydrophone (see Figure 1d) installed at a fixed position on the anchor. The hydrophone is a Colmar DG0330. It is omnidirectional, stereo (first channel has +26 dB gain and the other channel has +46 dB gain) to avoid signal saturation, and it works in the 5–90 kHz range. Its Received Voltage Response (RVR) is around −176 dB (re 1 V/μPa at 1 m) in the first channel and −156 dB (re 1 V/μPa at 1 m) in the other channel [15]. This hydrophone permits the calculation of the ToE of the autonomous ABs and facilitates bioacoustic studies.

The piezoceramic sensor installed inside each DOM is an acoustic receiver basically used to monitor the position of the DOM. It is in an aluminum capsule with the pre-amplifier board (see Figure 1b) glued to the glass of the DOM facing downwards. Its RVR is −160 ± 6 dB (re 1 V/μPa at 1 m) in the 10–70 kHz range [9].

#### 2.1.2. Positioning Process

The autonomous ABs emit automatically pulsed acoustic signals which are registered by the receivers. The KM3NeT clock generation system works with an internal signal-clock of 25 MHz [16], so it controls the time-stamp of recorded acoustic signals. The receivers record at 195.3125 kHz sample frequency (25 MHz/$2^{8_{bits}-1}$), so they can be used for receiving for signals with a frequency less than 100 kHz. The signals recorded are processed using a cross-correlation method in the frequency domain to determine the Time of Arrival (ToA) and identify its emitter. Assuming that the distance between emitter and receiver is known, and the sound speed gradient ($c_{sound}$) is estimated considering the environmental properties (the $c_{sound}$ along a DU is estimated between 1552.0 and 1563.8 m/s in ARCA, and between 1545.6 and 1549.0 m/s in ORCA [17]), it is possible to determine the ToE. From the ToA and the ToE values for three different emitters in the same receiver, it is possible to calculate the Time of Flight (ToF), so the distance, and do the triangulation to obtain the *x*, *y*, and *z* coordinates for every receiver [13,18].

### 2.2. Attitude and Heading Reference System (AHRS)

The Attitude and Heading Reference System (AHRS) in KM3NeT is focused on determining the orientation of every DOM. This information is important to determine where each Photomultiplier Tube (PMT) is and what its orientation is. It is provided by an accelerometer and a magnetometer in a PCB installed inside the DOMs. Combining their data, the rotation angles yaw (ψ), pitch (θ), and roll (ϕ) are determined and stored in the KM3NeT Data Base (DB). The boards used were designed by the KM3NeT Collaboration. The accuracy of the system is estimated to be smaller than 3.5 degrees [10].

**Figure 4:**
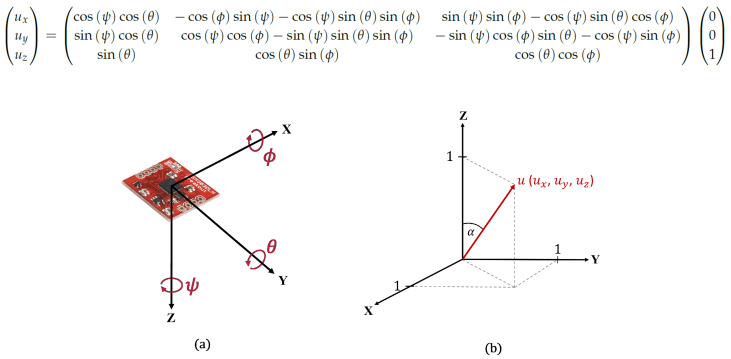
(**a**) The Printed Circuit Board (PCB) axis system in each DOM and yaw (ψ), pitch (θ), and roll (ϕ). (**b**) The absolute coordinate system for a DU uses a unitary point to locate the relative position of the DOMs of the DU.

The coordinate system of the Central Logic Board (CLB) of the DOM is defined relative to each DOM position (PCB position, see Figure 4a), so it is necessary to transform these coordinates to an absolute coordinate system for all DOMs (see Figure 4b). Once all DOM coordinates are defined relative to the same coordinate system, it will be possible to compare them and work with them as well. The following conversion matrix (obtained by the product of the three relevant rotation matrices [19]) is used to obtain the unitary *z-vector* of the DOM position in the new coordinate system:

The aim of this conversion is to determine the tilt (α) of the DOM position from the vertical of the DU-base. The tilt value (α, represents the zenith angle in Figure 4b) provides information about the status of the sea current. In the next section (Section 2.3) the importance of this value is explained. Therefore, it is possible to calculate the tilt (α) value from yaw, pitch, and roll values using Equation (1).

(1) $$\tan\left(\alpha \right)=\frac{\sqrt{u_{x}^{2}+u_{y}^{2}}}{u_{z}}$$

where α is the tilt value and $u_{x}$, $u_{y}$, and $u_{z}$ are the coordinates provided by the matrix to obtain the unitary *z-vector*.

### 2.3. Mechanical Model (MM)

The mechanical model (MM) in the DU Line Fit for KM3NeT is a set of mechanical equations used to determine the position and orientation of every DOM from sea current properties and the position of the DU-base. The MM uses mechanical constants ($MM_{const.}$) calculated from the mechanical properties of all items in a DU [18].

The MM for the KM3NeT DU was designed based on the line shape model of ANTARES, where the zenith angle (α, the tilt) at a given point of the line, is given by the ratio of the horizontal drag force Equation (3) and the vertical buoyancy forces Equation (4) summed over all line elements above the height *z* Equation (2) [8].

(2) $$tan\left(\alpha \right)=\frac{F(z)}{W(z)}=g\left(z\right)$$

where α is the zenith angle (represents the tilts of the DOM) at height *z* (from start of the ropes at 1.1 m to the maximum height of the DU), that depends on the horizontal force F(z) and vertical force W(z).

(3) $$F\left(z\right)=f\left(z\right)\;v^{2}=\left\{\left[\sum_{i=1}^{18}\left(f_{DOM}+f_{cable_{i}}\right)+f_{cable_{large}}\right]\left(\frac{h-z}{z}\right)+f_{top\;buoy}\right\}v^{2}$$

where F(z) represents the drag force at height *z* of the DU (with *h* the maximum height). It depends on the drag parameter *f* of all elements and on the sea current velocity *v*.

(4) $$W\left(z\right)=\left[\sum_{i=1}^{18}\left(W_{DOM}+W_{cable_{i}}\right)+W_{cable_{large}}\right]\left(\frac{h-z}{z}\right)+W_{top\;buoy}$$

where W(z) represents the vertical force that is dependent on the buoyancy force *W* of all elements along it.

In the MM calculations, two lengths for the cable of the DU are recognized (*cable* and $cable_{large}$) because the first part of the cable between DU-base and first DOM is considerably larger than the rest of the distances between DOMs. The sea current velocity is assumed to be the same along the entire line and the detector. By integration of g(z) over *z*, the total displacement between the item in height *z* can be obtained Equation (5) [18]:

(5) $$r\left(z\right)=\int_{0}^{z}g\left(z\right)\;dz=MM_{const.}\left(z\right)\cdot v^{2}$$

where r(z) represents the vertical displacement in the height *z* of the line considering the sea current velocity *v* and the mechanical constants ($MM_{const.}$) of DU parts.

In the MM it is considered that r(z) is determined by Equation (5). Therefore, by using the MM it is possible to predict the position of the DOMs along the line, given a sea current velocity value.

**Figure 5:**
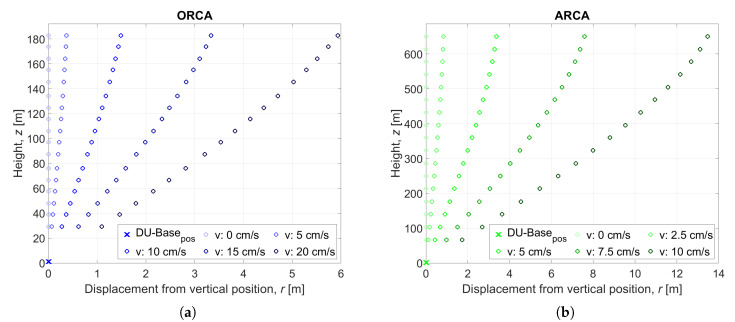
Mechanical model (MM) application to line shape in KM3NeT. (**a**) Line shape in ORCA for different sea currents values. (**b**) Line shape in ARCA for different sea current values.

The ORCA location is less deep than that of ARCA. Mean values of sea current velocity for ORCA are around 5–7 cm/s, while in ARCA the values are around 2–3 cm/s. Figure 5 shows the application of MM in the reconstruction of the line shape for both cases, in intervals of sea current velocities from low to critical values.

**Figure 6:**
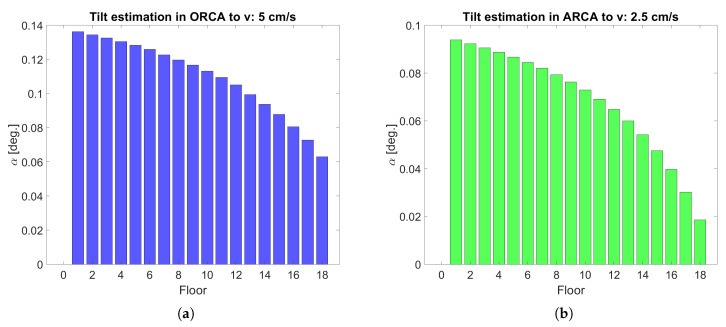
Tilt value from MM application for the DOMs in a DU (labeled Floor, with Floor 1 the lowest DOM in the DU) using representative sea current values in ORCA and ARCA. (**a**) Tilt in ORCA for a typical sea current of 5 cm/s. (**b**) Tilt in ARCA for a typical sea current of 2.5 cm/s.

Since the tilt value (α) of each DOM is proportional to the increase of displacement from its vertical position (*r*), it is possible to observe the biggest tilt value on of the lowest DOM in the DU (labeled Floor 1) because of $cable_{large}$. In Figure 6 the inclination of each DOM is shown derived from the tilt value measured at the lowest DOM.

## 3. The Detection Unit Line Fit

The DU Line Fit is the process that combines the data from APS and AHRS (as input parameters from the same DU) with the MM to determine the real position of the DOMs every 10 min. Once the input data is filtered from anomalous values and averaged, the shape of the DU is reconstructed using Equation (5) with the effective sea current velocity (*v*) as fit parameter. Then, the position and orientation of every DOM in the DU is obtained. Therefore, the line shape fit is applied for each DU, although the effective sea current direction and velocity values will be expected to be similar in all DUs of the same detector (these values will be controlled remotely). The procedure is as follows.

KM3NeT data are stored in RUN files, usually during a time window of four hours. The APS data are taken and processed every 5 s saving the value of the peak amplitude in the cross-correlation signal together with the timestamp of this maximum. The APS can distinguish if it is an AB emission or if it corresponds to noise. Then, the APS data are used to position each receiver (*x*, *y*, and *z* values) according to the valid time detection of the acoustic signals from the ABs. The AHRS data (yaw, pitch, and roll values) are recorded every 3 s. The DU Line Fit uses the mean values of the input parameters every 10 min (during 10 min the change in the position and orientation can be neglected since it is slow motion). In fact, the DU Line Fit will be usually applied once the RUN is finished, not as a real-time process.

The DU Line Fit takes the mean of the raw positioning and orientation values in a time window of 10 min as input. Studying the DOM positions (*x*, *y*, and *z* values) relative to the lowest DOM in the top view plane (bi-dimensional), it is easy to predict an effective sea current direction. Using the MM from APS and AHRS data it is possible to determine an effective sea current velocity. Then, the reconstruction of the DU shape is possible.

**Figure 7:**
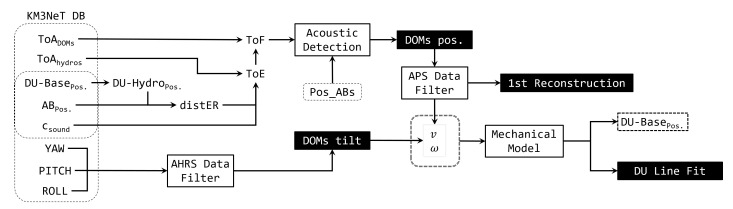
DU Line Fit block diagram. This scheme shows the steps in the implementation of the DU Line Fit in KM3NeT detectors.

In summary (see Figure 7), the APS registers the ToA of AB signals in the receivers. It uses the ToA of the hydrophones ($ToA_{hydros}$) and the distance between the hydrophones and the emitters (distER) to estimate the ToE for each autonomous AB. With these ToEs and the sound speed gradient ($c_{sound}$), the ToF is calculated ($ToF=ToA-ToE_{AB}$). Once the ToF and the autonomous ABs position (Pos_ABs) are known, the acoustic detection system can position all acoustic receivers on the detector. At this point, in low sea current periods, it is possible do a first reconstruction using an interpolation function in 3D to obtain the position of the DOMs, smooth the fit and position. The AHRS saves the yaw, pitch, and roll value of each DOM. Analyzing these data it is possible to calculate the tilt value of the DOMs. Using the MM constants and equations, combining the position and tilt of the DOMs it is possible to assume effective sea current properties (*v* & ω) to reconstruct the shape of the DU.

## 4. Conclusions

The DU Line Fit allows for the conversion from raw data from acoustic and AHRS sensors to precisely determine the position and orientation of the DOMs in a DU. The accurate monitoring of the position ( 10 cm) and orientation (a few degrees) achieved by the mechanical model reconstruction from the input of the sensors is important for a correct reconstruction of detected events.

In this paper, we have described the systems and shown the use of the DU Line Fit system for measuring the line shape of the DUs in the KM3NeT telescope.

## Abbreviations

The following abbreviations are used in this manuscript:

AB	Acoustic Beacon
AHRS	Attitude and Heading Reference System
APS	Acoustic Positioning System
CLB	Central Logic Board
DB	Data Base
DOM	Digital Optical Module
DU	Detection Unit
MM	Mechanical model
PCB	Printed Circuit Board
PMT	Photomultiplier Tube
RVR	Received Voltage Response
SPL	Sound Pressure Level
ToA	Time of Arrival
ToE	Time of Emission
ToF	Time of Flight

## Appendix A. KM3NeT Collaboration

*–Full Author List–*

S. Aiello${}^{a}$, A. Albert${}^{bd,b}$, S. Alves Garre${}^{c}$, Z. Aly${}^{d}$, A. Ambrosone${}^{e,f}$, F. Ameli${}^{g}$, M. Andre${}^{h}$, G. Androulakis${}^{i}$, M. Anghinolfi${}^{j}$, M. Anguita${}^{k}$, G. Anton${}^{l}$, M. Ardid${}^{m}$, J. Aublin${}^{n}$, C. Bagatelas${}^{i}$, G. Barbarino${}^{e,f}$, B. Baret${}^{n}$, S. Basegmez du Pree${}^{o}$, M. Bendahman${}^{p}$, E. Berbee${}^{o}$, A. M. van den Berg${}^{q}$, V. Bertin${}^{d}$, S. Biagi${}^{r}$, M. Bissinger${}^{l}$, M. Boettcher${}^{s}$, M. Bou Cabo${}^{t}$, J. Boumaaza${}^{p}$, M. Bouta${}^{u}$, M. Bouwhuis${}^{o}$, C. Bozza${}^{v}$, H.Brânzaş${}^{w}$, R. Bruijn${}^{o,x}$, J. Brunner${}^{d}$, E. Buis${}^{y}$, R. Buompane${}^{e,z}$, J. Busto${}^{d}$, B. Caiffi${}^{j}$, D. Calvo${}^{c}$, A. Capone${}^{aa,g}$, V. Carretero${}^{c}$, P. Castaldi${}^{ab,ac}$, S. Celli${}^{aa,g,be}$, M. Chabab${}^{ad}$, N. Chau${}^{n}$, A. Chen${}^{ae}$, S. Cherubini${}^{r,af}$, V. Chiarella${}^{ag}$, T. Chiarusi${}^{ab}$, M. Circella${}^{ah}$, R. Cocimano${}^{r}$, J. A. B. Coelho${}^{n}$, A. Coleiro${}^{n}$, M. Colomer Molla${}^{n,c}$, S. Colonges${}^{n}$, R. Coniglione${}^{r}$, I. Corredoira${}^{c}$, P. Coyle${}^{d}$, A. Creusot${}^{n}$, G. Cuttone${}^{r}$, A. D’Onofrio${}^{e,z}$, R. Dallier${}^{ai}$, M. De Palma${}^{ah,aj}$, M. Di Marino${}^{ak}$, I. Di Palma${}^{aa,g}$, A. F. Díaz${}^{k}$, D. Diego-Tortosa${}^{m}$, C. Distefano${}^{r}$, A. Domi${}^{j,d,al}$, C. Donzaud${}^{n}$, D. Dornic${}^{d}$, M. Dörr${}^{am}$, D. Drouhin${}^{bd,b}$, T. Eberl${}^{l}$, A. Eddyamoui${}^{p}$, T. van Eeden${}^{o}$, D. van Eijk${}^{o}$, I. El Bojaddaini${}^{u}$, D. Elsaesser${}^{am}$, A. Enzenhöfer${}^{d}$, V. Espinosa${}^{m}$, P. Fermani${}^{aa,g}$, G. Ferrara${}^{r,af}$, M. D. Filipović${}^{an}$, F. Filippini${}^{ab,ao}$, L. A. Fusco${}^{d}$, O. Gabella${}^{ap}$, T. Gal${}^{l}$, A. Garcia Soto${}^{o}$, F. Garufi${}^{e,f}$, Y. Gatelet${}^{n}$, N. Geißelbrecht${}^{l}$, L. Gialanella${}^{e,z}$, E. Giorgio${}^{r}$, S. R. Gozzini${}^{l}$, R. Gracia${}^{o}$, K. Graf${}^{l}$, D. Grasso${}^{aq}$, G. Grella${}^{ak}$, C. Guidi${}^{j,al}$, S. Hallmann${}^{l}$, H. Hamdaoui${}^{p}$, H. van Haren${}^{ar}$, A. Heijboer${}^{o}$, A. Hekalo${}^{am}$, J. J. Hernández-Rey${}^{c}$, J. Hofestädt${}^{l}$, F. Huang${}^{as}$, W. Idrissi Ibnsalih${}^{e,z}$, A. Ilioni${}^{n}$, G. Illuminati${}^{n,c}$, C. W. James${}^{at}$, M. de Jong${}^{o}$, P. de Jong${}^{o,x}$, B. J. Jung${}^{o}$, M. Kadler${}^{am}$, P. Kalaczyński${}^{au}$, O. Kalekin${}^{l}$, U. F. Katz${}^{l}$, N. R. Khan Chowdhury${}^{c}$, G. Kistauri${}^{av}$, F. van der Knaap${}^{y}$, E. N. Koffeman${}^{o,x}$, P. Kooijman${}^{x,bf}$, A. Kouchner${}^{n,aw}$, M. Kreter${}^{s}$, V. Kulikovskiy${}^{j}$, R. Lahmann${}^{l}$, G. Larosa${}^{r}$, R. Le Breton${}^{n}$, O. Leonardi${}^{r}$, F. Leone${}^{r,af}$, E. Leonora${}^{a}$, G. Levi${}^{ab,ao}$, M. Lincetto${}^{d}$, M. Lindsey Clark${}^{n}$, T. Lipreau${}^{ai}$, F. Longhitano${}^{a}$, D. Lopez-Coto${}^{ax}$, L. Maderer${}^{n}$, J. Mańczak${}^{c}$, K. Mannheim${}^{am}$, A. Margiotta${}^{ab,ao}$, A. Marinelli^e^, C. Markou${}^{i}$, L. Martin${}^{ai}$, J. A. Martínez-Mora${}^{m}$, A. Martini${}^{ag}$, F. Marzaioli${}^{e,z}$, S. Mastroianni^e^, S. Mazzou${}^{ad}$, K. W. Melis${}^{o}$, G. Miele${}^{e,f}$, P. Migliozzi^e^, E. Migneco${}^{r}$, P. Mijakowski${}^{au}$, L. S. Miranda${}^{ay}$, C. M. Mollo^e^, M. Morganti${}^{aq,bg}$, M. Moser${}^{l}$, A. Moussa${}^{u}$, R. Muller${}^{o}$, D. Muñoz Pérez${}^{c}$, M. Musumeci${}^{r}$, L. Nauta${}^{o}$, S. Navas${}^{ax}$, C. A. Nicolau${}^{g}$, B. Ó Fearraigh${}^{o,x}$, M. O’Sullivan${}^{at}$, M. Organokov${}^{as}$, A. Orlando${}^{r}$, J. Palacios González${}^{c}$, G. Papalashvili${}^{av}$, R. Papaleo${}^{r}$, C. Pastore${}^{ah}$, A. M. Păun${}^{w}$, G. E. Păvălaş${}^{w}$, C. Pellegrino${}^{ao,bh}$, M. Perrin-Terrin${}^{d}$, V. Pestel${}^{o}$, P. Piattelli${}^{r}$, C. Pieterse${}^{c}$, K. Pikounis${}^{i}$, O. Pisanti${}^{e,f}$, C. Poirè${}^{m}$, V. Popa${}^{w}$, T. Pradier${}^{as}$, G. Pühlhofer${}^{az}$, S. Pulvirenti${}^{r}$, O. Rabyang${}^{s}$, F. Raffaelli${}^{aq}$, N. Randazzo${}^{a}$, S. Razzaque${}^{ay}$, D. Real${}^{c}$, S. Reck${}^{l}$, G. Riccobene${}^{r}$, M. Richer${}^{as}$, S. Rivoire${}^{ap}$, A. Rovelli${}^{r}$, F. Salesa Greus${}^{c}$, D. F. E. Samtleben${}^{o,ba}$, A. Sánchez Losa${}^{ah}$, M. Sanguineti${}^{j,al}$, A. Santangelo${}^{az}$, D. Santonocito${}^{r}$, P. Sapienza${}^{r}$, J. Schnabel${}^{l}$, J. Schumann${}^{l}$, J. Seneca${}^{o}$, I. Sgura${}^{ah}$, R. Shanidze${}^{av}$, A. Sharma${}^{bb}$, F. Simeone${}^{g}$, A.Sinopoulou${}^{i}$, B. Spisso${}^{ak,e}$, M. Spurio${}^{ab,ao}$, D. Stavropoulos${}^{i}$, J. Steijger${}^{o}$, S. M. Stellacci${}^{ak,e}$, M. Taiuti${}^{j,al}$, Y. Tayalati${}^{p}$, E. Tenllado${}^{ax}$, T. Thakore${}^{c}$, H. Thiersen${}^{s}$, S. Tingay${}^{at}$, E. Tzamariudaki${}^{i}$, D. Tzanetatos${}^{i}$, V. Van Elewyck${}^{n,aw}$, G. Vasileiadis${}^{ap}$, F. Versari${}^{ab,ao}$, S. Viola${}^{r}$, D. Vivolo${}^{e,f}$, G. de Wasseige${}^{n}$, J. Wilms${}^{bc}$, R. Wojaczyński${}^{au}$, E. de Wolf${}^{o,x}$, S. Zavatarelli${}^{j}$, A. Zegarelli${}^{aa,g}$, D. Zito${}^{r}$, J. D. Zornoza${}^{c}$, J. Zúñiga${}^{c}$, N. Zywucka${}^{s}$

${}^{a}$INFN, Sezione di Catania, Via Santa Sofia 64, 95123 Catania, Italy

${}^{b}$IN2P3, IPHC, 23 rue du Loess, 67037 Strasbourg, France

${}^{c}$IFIC - Instituto de Física Corpuscular (CSIC - Universitat de València), c/Catedrático José Beltrán, 2, 46980 Paterna, Valencia, Spain

${}^{d}$Aix Marseille Univ, CNRS/IN2P3, CPPM, Marseille, France

^e^INFN, Sezione di Napoli, Complesso Universitario di Monte S. Angelo, Via Cintia ed. G, 80126 Napoli, Italy

${}^{f}$Università di Napoli “Federico II”, Dip. Scienze Fisiche “E. Pancini”, Complesso Universitario di Monte S. Angelo, Via Cintia ed. G, 80126 Napoli, Italy

${}^{g}$INFN, Sezione di Roma, Piazzale Aldo Moro 2, 00185 Roma, Italy

${}^{h}$Universitat Politècnica de Catalunya, Laboratori d’Aplicacions Bioacústiques, Centre Tecnològic de Vilanova i la Geltrú, Avda. Rambla Exposició, s/n, 08800 Vilanova i la Geltrú, Spain

${}^{i}$NCSR Demokritos, Institute of Nuclear and Particle Physics, Ag. Paraskevi Attikis, 15310 Athens, Greece

${}^{j}$INFN, Sezione di Genova, Via Dodecaneso 33, 16146 Genova, Italy

${}^{k}$University of Granada, Dept. of Computer Architecture and Technology/CITIC, 18071 Granada, Spain

${}^{l}$Friedrich-Alexander-Universität Erlangen-Nürnberg, Erlangen Centre for Astroparticle Physics, Erwin-Rommel-Straße 1, 91058 Erlangen, Germany

${}^{m}$Universitat Politècnica de València (UPV)—Institut d’Investigació per a la Gestió Integrada de les Zones Costaneres (IGIC), Paranimf 1, 46730 Grau de Gandia (València), Spain

${}^{n}$Université de Paris, CNRS, Astroparticule et Cosmologie, F-75013 Paris, France

${}^{o}$Nikhef, National Institute for Subatomic Physics, PO Box 41882, 1009 DB Amsterdam, Netherlands

${}^{p}$University Mohammed V in Rabat, Faculty of Sciences, 4 av. Ibn Battouta, B.P. 1014, R.P. 10000 Rabat, Morocco

${}^{q}$KVI-CART University of Groningen, Groningen, the Netherlands

${}^{r}$INFN, Laboratori Nazionali del Sud, Via S. Sofia 62, 95123 Catania, Italy

${}^{s}$North-West University, Centre for Space Research, Private Bag X6001, Potchefstroom, 2520 South Africa

${}^{t}$Instituto Español de Oceanografía, Unidad Mixta IEO-UPV, C/ Paranimf, 1, 46730 Gandia, Spain

${}^{u}$University Mohammed I, Faculty of Sciences, BV Mohammed VI, B.P. 717, R.P. 60000 Oujda, Morocco

${}^{v}$Università di Salerno e INFN Gruppo Collegato di Salerno, Dipartimento di Matematica, Via Giovanni Paolo II 132, 84084 Fisciano, Italy

${}^{w}$ISS, Atomistilor 409, RO-077125 Măgurele, Romania

${}^{x}$University of Amsterdam, Institute of Physics/IHEF, PO Box 94216, 1090 GE Amsterdam, Netherlands

${}^{y}$TNO, Technical Sciences, PO Box 155, 2600 AD Delft, The Netherlands

${}^{z}$Università degli Studi della Campania "Luigi Vanvitelli", Dipartimento di Matematica e Fisica, viale Lincoln 5, 81100 Caserta, Italy

${}^{aa}$Università La Sapienza, Dipartimento di Fisica, Piazzale Aldo Moro 2, 00185 Roma, Italy

${}^{ab}$INFN, Sezione di Bologna, v.le C. Berti-Pichat, 6/2, 40127 Bologna, Italy

${}^{ac}$Università di Bologna, Dipartimento di Ingegneria dell’Energia Elettrica e dell’Informazione Guglielmo Marconi, v.le C. Berti-Pichat, 6/2, 40127 Bologna, Italy

${}^{ad}$Cadi Ayyad University, Physics Department, Faculty of Science Semlalia, Av. My Abdellah, P.O.B. 2390, 40000 Marrakech, Morocco

${}^{ae}$University of the Witwatersrand, School of Physics, Private Bag 3, Johannesburg, Wits 2050 South Africa

${}^{af}$Università di Catania, Dipartimento di Fisica e Astronomia “Ettore Majorana”, Via Santa Sofia 64, 95123 Catania, Italy

${}^{ag}$INFN, LNF, Via Enrico Fermi, 40, 00044 Frascati, Italy

${}^{ah}$INFN, Sezione di Bari, Via Amendola 173, 70126 Bari, Italy

${}^{ai}$Subatech, IMT Atlantique, IN2P3-CNRS, Université de Nantes, 4 rue Alfred Kastler - La Chantrerie, BP 20722 44307 Nantes, France

${}^{aj}$University of Bari, Via Amendola 173, 70126 Bari, Italy

${}^{ak}$Università di Salerno e INFN Gruppo Collegato di Salerno, Dipartimento di Fisica, Via Giovanni Paolo II 132, 84084 Fisciano, Italy

${}^{al}$Università di Genova, Via Dodecaneso 33, 16146 Genova, Italy

${}^{am}$University Würzburg, Emil-Fischer-Straße 31, 97074 Würzburg, Germany

${}^{an}$Western Sydney University, School of Computing, Engineering and Mathematics, Locked Bag 1797, Penrith, NSW 2751, Australia

${}^{ao}$Università di Bologna, Dipartimento di Fisica e Astronomia, v.le C. Berti-Pichat, 6/2, 40127 Bologna, Italy

${}^{ap}$Laboratoire Univers et Particules de Montpellier, Place Eugène Bataillon - CC 72, Montpellier Cédex 05, 34095 France

${}^{aq}$INFN, Sezione di Pisa, Largo Bruno Pontecorvo 3, 56127 Pisa, Italy

${}^{ar}$NIOZ (Royal Netherlands Institute for Sea Research) and Utrecht University, PO Box 59, Den Burg, 1790 AB Texel, the Netherlands

${}^{as}$Université de Strasbourg, CNRS IPHC UMR 7178, 23 rue du Loess, 67037 Strasbourg, France

${}^{at}$International Centre for Radio Astronomy Research, Curtin University, Bentley, WA 6102, Australia

${}^{au}$National Centre for Nuclear Research, 02-093 Warsaw, Poland

${}^{av}$Tbilisi State University, Department of Physics, 3, Chavchavadze Ave., 0179 Tbilisi, Georgia

${}^{aw}$Institut Universitaire de France, 1 rue Descartes, 75005 Paris, France

${}^{ax}$University of Granada, Dpto. de Física Teórica y del Cosmos & C.A.F.P.E., 18071 Granada, Spain

${}^{ay}$University of Johannesburg, Department Physics, PO Box 524, Auckland Park, 2006 South Africa

${}^{az}$Eberhard Karls Universität Tübingen, Institut für Astronomie und Astrophysik, Sand 1, 72076 Tübingen, Germany

${}^{ba}$Leiden University, Leiden Institute of Physics, PO Box 9504, 2300 RA Leiden, Netherlands

${}^{bb}$Università di Pisa, Dipartimento di Fisica, Largo Bruno Pontecorvo 3, 56127 Pisa, Italy

${}^{bc}$Friedrich-Alexander-Universität Erlangen-Nürnberg, Remeis Sternwarte, Sternwartstraße 7, 96049 Bamberg, Germany

${}^{bd}$Université de Strasbourg, Université de Haute Alsace, GRPHE, 34, Rue du Grillenbreit, 68008 Colmar, France

${}^{be}$Gran Sasso Science Institute, GSSI, Viale Francesco Crispi 7, 67100 L’Aquila, Italy

${}^{bf}$Utrecht University, Department of Physics and Astronomy, PO Box 80000, 3508 TA Utrecht, The Netherlands

${}^{bg}$Accademia Navale di Livorno, Viale Italia 72, 57100 Livorno, Italy

${}^{bh}$INFN, CNAF, v.le C. Berti-Pichat, 6/2, 40127 Bologna, Italy
